# Toolbox for Research, or how to facilitate a central data management in small-scale research projects

**DOI:** 10.1186/s12967-018-1390-1

**Published:** 2018-01-25

**Authors:** Martin Bialke, Henriette Rau, Oliver C. Thamm, Ronny Schuldt, Peter Penndorf, Arne Blumentritt, Robert Gött, Jens Piegsa, Thomas Bahls, Wolfgang Hoffmann

**Affiliations:** 1 Department Epidemiology of Health Care and Community Health, Institute for Community Medicine, University Medicine Greifswald, Ellernholzstr. 1-2, 17487 Greifswald, Germany; 2 Klinik für Plastische und Ästhetische Chirurgie, Sana-Krankenhaus Gerresheim, Gräulinger Straße 120, 40625 Düsseldorf, Germany

**Keywords:** Data management, Epidemiological research, Pseudonymization, Research repository, Data dictionary, ECRF

## Abstract

**Background:**

In most research projects budget, staff and IT infrastructures are limiting resources. Especially for small-scale registries and cohort studies professional IT support and commercial electronic data capture systems are too expensive. Consequently, these projects use simple local approaches (e.g. Excel) for data capture instead of a central data management including web-based data capture and proper research databases. This leads to manual processes to merge, analyze and, if possible, pseudonymize research data of different study sites.

**Results:**

To support multi-site data capture, storage and analyses in small-scall research projects, corresponding requirements were analyzed within the MOSAIC project. Based on the identified requirements, the Toolbox for Research was developed as a flexible software solution for various research scenarios. Additionally, the Toolbox facilitates data integration of research data as well as metadata by performing necessary procedures automatically. Also, Toolbox modules allow the integration of device data. Moreover, separation of personally identifiable information and medical data by using only pseudonyms for storing medical data ensures the compliance to data protection regulations. This pseudonymized data can then be exported in SPSS format in order to enable scientists to prepare reports and analyses.

**Conclusions:**

The Toolbox for Research was successfully piloted in the German Burn Registry in 2016 facilitating the documentation of 4350 burn cases at 54 study sites. The Toolbox for Research can be downloaded free of charge from the project website and automatically installed due to the use of Docker technology.

## Background

The capture, processing, storage and usage of research data in compliance with data protection requirements has become a focus in epidemiological research projects [1]. High quality research data is the basis for reliable analyses and valid answers to epidemiological research questions. The data collection process itself can be a major source of error, however the requirement to improve quality and technical processes can raise barriers against the initiation of epidemiological studies [2]. Therefore, a priori planning of a comprehensive data management is one of the core elements in the design and implementation of population studies. In the context of increasingly large and multi-site research projects, e.g. the German National Cohort (NAKO) [3] as well as studies and registries of the German Centre for Cardiovascular Disease (DZHK) [4], the requirements for a comprehensive data management include the following [1]:

Since registries and cohort studies differ with respect to their application scenario, the methods of data capture (e.g. eCRF), the data dictionary, the integration of laboratory or medical devices, quality assurance methods and export formats have to be adapted to the studies respectively. Therefore, several options and software solutions are available to researchers when planning the implementation of an electronic data capture using eCRFs. For example, researchers can decide to use commercial (e.g. TeleForm), open-source or free of charge (OpenClinica, tranSMART or REDCap) solutions or to develop individual software solutions on their own with high personal efforts. However, many small-scale research projects may not be able to afford commercial or self-implemented EDC systems. Especially smaller registries and cohort studies with limited information technology (IT) resources in terms of IT knowledge, staff and infrastructure can usually not afford the necessary technical, organizational and staff resources [1, 2]. Thus, additional requirements exist in small-scale research projects (see Table 2) to select suitable tools. Presently, small-scale research projects often use general-purpose applications developed for office use, e.g. spreadsheet, which can easily compromise data quality and safety [6].

As an example, the German Burn Registry [7], which aims at improving the management of patients with burn injuries based on experiences from current treatment, applied data capture approaches using MS Excel as an alternative to comprehensive EDC systems, before the development of the “Toolbox for Research”. Consequently, data from participating local registry sites are manually merged at a central location for annual analysis. In this case, pseudonymization is most likely conducted manually, if at all. As one consequence, an integration of additional information, e.g. from devices, into a central data repository is almost impossible. A web-based software solution, which implements the mentioned requirements for a comprehensive data management (see Tables 1, 2), could be used to technically upgrade the German Burn Registry [7], minimize current documentation efforts in specialized intensive care units for patients and to support a better quality assurance and scientific evaluation as well as the development of quality guidelines.

**Table 1:** List of requirements for a comprehensive data management based on [1]

No.	Requirement
1	Development of a data dictionary (DD) and electronic Case Report Forms (eCRF) for data capture
2	Generation and provision of web-based questionnaires in the form of eCRFs for central data collection
3	Separation of personally identifiable information (PII) [5] as well as medical research data (MDAT) including the generation of pseudonyms as early as possible within data processing
4	Separate storage of metadata and pseudonymized MDAT
5	ETL-processes: extraction of relevant data from connected data sources, transformation of the data to a uniform (internal) data format, the enrichment of metadata and the loading of the enriched data into the research data repository
6	Export and transfer of uniform, pseudonymized data in at least one standardized format (e.g. SPSS or SAS) for data analysis and/or quality assurance
7	Ensuring the possibility of authentication (managing users as well as roles and/ or rights) as well as study process and site management

**Table 2:** List of additional requirements concerning data management for small-scale research projects based on [2]

No.	Requirement
a1	Open-source solution, free or with very low costs
a2	Easy download and installation and/ or web-based data capture
a3	Useful range of functionality, e.g. extensibility with additional modules like interfaces for integrating additional data sources
a4	Adequate community support
a5	Possibility for low-level training
a6	User-friendliness and intuitive usability, e.g. easy design of eCRFs
a7	Sufficient documentation
a8	Management of organizational and technical processes to use and to provide access to the pseudonymized research data

In order to support researchers as well as the scientific community, and to address a large number of research projects in different scenarios, a flexible software solution is needed. This software solution should address all requirements for a comprehensive data management in research (see Table 1) and also consider the additional requirements of small-scale research projects (see Table 2). Consequently, the software solution must be freely accessible, free of charge and, therefore, based on open-source solutions. Additionally, it should be easy to apply and suitable for small but heterogeneous research projects. For this purpose, the “Toolbox for Research” was developed within the MOSAIC project [1] by the Institute for Community Medicine of the University Medicine Greifswald.

The Toolbox for Research addresses researchers with limited IT knowledge and resources. Therefore, the Toolbox minimizes the efforts for installing, configuring and operating the software solution. This includes basic technical support for the deployment of the system with an automatic installation routine. Additionally, extensive user manuals including installation and configuration, as well as guidelines and templates to develop the necessary DD and eCRFs have to be provided.

As a proof of concept, the Toolbox for Research is utilised to re-implement the German Burn Registry [7].

## Methods

**Fig. 1:**
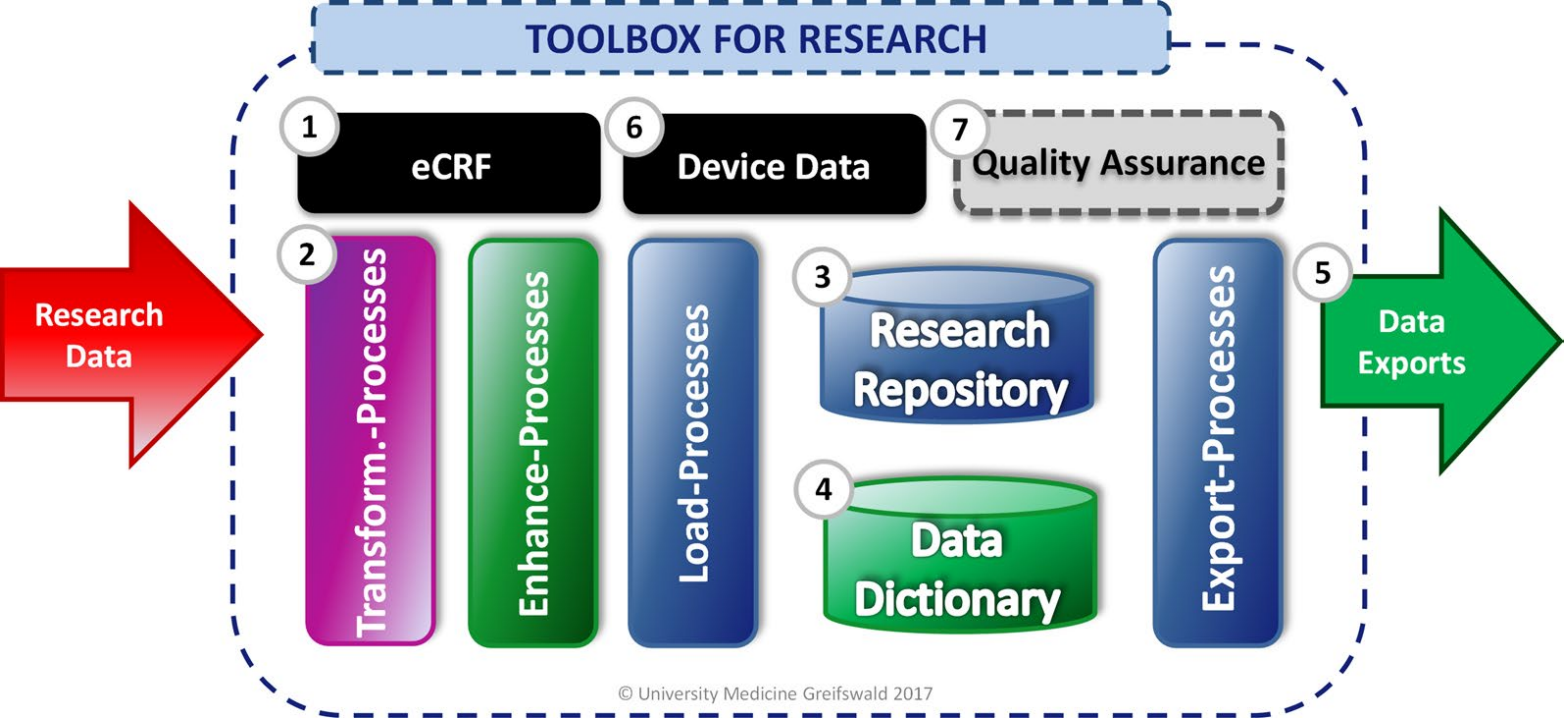
The functionalities of the modular Toolbox for Research can easily be extended with additional modules e.g. for quality assurance

The Toolbox for Research adopts a modular approach for data management. As a result, incoming research data are being processed step-by-step (see Fig. 1) in order to provide a uniform export format for subsequent data analysis:

1. The Toolbox for Research provides web-based eCRFs for a central data capture.
2. The captured data are automatically processed by integrated ETL processes (data integration).
3. Research data is separated from metadata and stored within a research repository (R2).
4. Additional metadata describing precisely the context of captured research data are stored strictly separated from the research data within a comprehensive data dictionary (D2).
5. Continuous data exports of the primary data are generated in the desired output format (for instance, SPSS) and centrally provided for authorized download.
6. The Toolbox processes research data from individual devices and automatically integrates it using the very same internal processes.
7. Additional functions, such as a module for daily quality assurance, can be integrated if necessary.

### Developing a data dictionary and generating eCRFs

The development of a DD and eCRF for data capture are essential aspects of a comprehensive data management within epidemiological research projects.

Within the Toolbox for Research, guidelines and templates for developing a data dictionary [8] as well as eCRFs [9] are provided in English and German language. Additionally, a data dictionary template (provided as Excel spreadsheet) allows the researcher to develop a data dictionary manually according to the data dictionary guideline without the need of specialized IT-knowledge. The template then becomes the basis for the generation of web-based data entry forms with the help of OpenClinica [10].

To be able to address several requirements of data management (see Tables 1, 2), the Toolbox for Research comes with a fully integrated instance of OpenClinica, which also allows the generation of web-based questionnaires and metadata descriptions. Franklin et al. evaluated three different open-source or free of charge software tools—OpenClinica, REDCap and Catalyst Web Tools—for small-scale research projects. According to these authors OpenClinica provides a wide array of functions to design complex eCRFs, since it is developed exclusively as EDC system. Additionally, OpenClinica enables the administration of sites, their eCRFs and users as well as the allocation of appropriate roles and rights. Although OpenClinica provided the most extended functionality, it was only second in the evaluation of Franklin et al. due to lack of easy to understand documentation at the time of the evaluation. The preferred EDC software REDCap, however, is not open-source [2].

OpenClinica is used within the Toolbox for Research, because of its active developer and user community and its nowadays extensive documentation. OpenClinica is also well-established within clinical research and provides not only user and site management but also numerous security features like authentication. Additionally, it allows the development of complex eCRFs, and automatization as well as integration of other systems via several web interfaces. Data exports from OpenClinica in CDISC-ODM format are automatically processed and integrated into the Toolbox-internal research repository and metadata dictionary.

### Separating research information and metadata

The Toolbox for Research provides a modular and readily re-usable study database with separate databases (see Fig. 1) for both research data in a research repository (R2) as well as metadata in an integrated data dictionary (D2). The underlying data model for this approach is used within the GANI_MED project [11]. Especially for the use in smaller cohort studies and registries, storage of research and metadata should be simple and at the same time flexible to permit the mapping of information during runtime.

Advantages of this innovative metadata approach [12] are an unlimited depth of hierarchical elements, the extensibility of element properties without the need to apply structural changes. Additionally, it is possible to map and illustrate relationships between different data elements as well as to define additional tree or network structures (such as graphs) if needed. To this extent, metadata is mapped in an entity relationship model with separate databases to store research and metadata. Moreover, data can be converted into common metadata models (CDISC-ODM, XML, etc.). Although this metadata approach facilitates nowadays cohort studies and smaller registries it is not limited to applications within an epidemiological context. It is also applicable to other eCRF-based use cases, e.g. capturing medical data of common diseases, like cancer or diabetes, as well as patient-reported outcomes, e.g. regarding depression or pain.

### Simplified processing through automatization

To be able to benefit from the advantages of the flexible research repository and data dictionary, the Toolbox supports an automated data integration to perform the necessary ETL-procedures. The data integration module (OCDI module) processes and validates incoming data (containing research data as well as metadata) in predefined formats (CDISC-ODM, CSV or ZIP). The validation process is based on predefined metadata definitions (for device data) as well as automatically learned metadata (for OpenClinica forms data). Invalid research data, in terms of unknown data, data types or invalide values, halts the integration process and requires manual interaction. All functionalities of the data integration module are provided via web-services and can be used to process OpenClinica data exports as well as external device data.

As depicted in Fig. 1, incoming data are transformed from the input format to an internal uniform data format as the first step of data import.

In a second step research data is enriched with metadata references to corresponding metadata if necessary. Such corresponding metadata can consist of variable descriptions, units, code lists and, if applicable, other characteristics of the variables like range, limits or qualitative missings. In contrast to transformation processes, which should not have any knowledge of the underlying metadata, the enhancing processes must be able to query the study`s metadata repository within the integrated data dictionary. If incoming raw data consist of aggregated research and metadata, an integrated converter in the Toolbox of Research separates both data types.

In the last step, load processes store the enriched research data in the research repository.

### Export of pseudonymized research data

In most cases scientists prefer a pragmatic approach to access data that does not require knowledge of technical details about data integration. In the Toolbox it is not necessary to access both databases directly to compile the needed data for analyses. Rather, research data and corresponding metadata are automatically aggregated and exported to a uniform data format for monitoring as well as research purposes and centrally provided to the scientists for download. As an example, data exports from the Toolbox for Research are converted in a ready to use SPSS format (*.sps and *.dat files) in order to enable scientists to prepare reports and analyses based on the exported data without any further transformation. Importing the generated data export file into the alternative open-source software package R [13] for statistical analyses is also possible.

### Ensuring data protection and pseudonymization

The European General Data Protection Regulation emphasizes the separation of PII and MDAT.

The Toolbox for Research provides the necessary pseudonymization services with the help of the modular web service gPAS (generic pseudonym administration service) [14] . gPAS allows to generate multiple pseudonyms, e.g. for local patient identifiers or case numbers. Pseudonyms can be easily configured and individually designed to match study-specific requirements. gPAS enables to pseudonymize and de-pseudonymize data records as well as to validate pseudonyms. With the help of pseudonym domains gPAS allows for specific pseudonym generation for e.g. different study sites, various devices or for consecutive exports for data analysis.

**Fig. 2:**
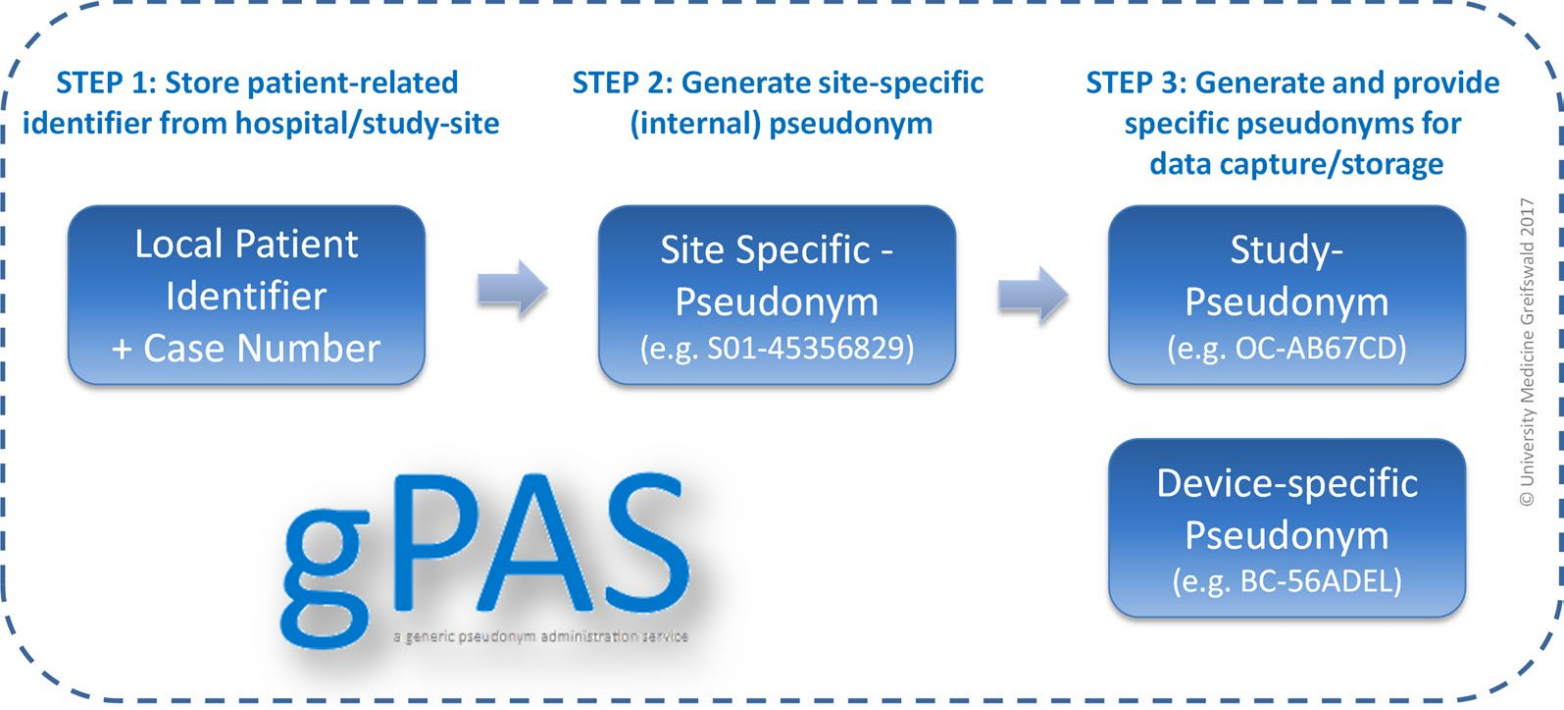
The integrated pseudonymization service gPAS provides the necessary pseudonyms

In the Toolbox for Research, gPAS generates a site-specific pseudonym for a given combination of local patient identifier and case number. Afterwards, an additional pseudonym for web-based data capture with OpenClinica (study pseudonym) and device-specific pseudonyms (e.g. for BurnCase3D [15] in the context of the German Burn Registry) are generated and mapped to the corresponding parent pseudonym (see Fig. 2).

The Toolbox for Research does currently not recognize duplicates of patients or study participants. As a consequence, it cannot ensure unambiguous identification of individuals. Patient ID and case number are stored in a pseudonymized form. All personally identifiable information (e.g. first name, surname and address) stay locally in the clinical system and are not transmitted to, or stored within the Toolbox.

Keeping track of a patient can be performed by any appropriate parameter, e.g. a previously assigned pseudonym that is used in the eCRF. To be compliant with data protection requirements, this ID has to be managed and audited autonomously at the study site.

**Fig. 3:**
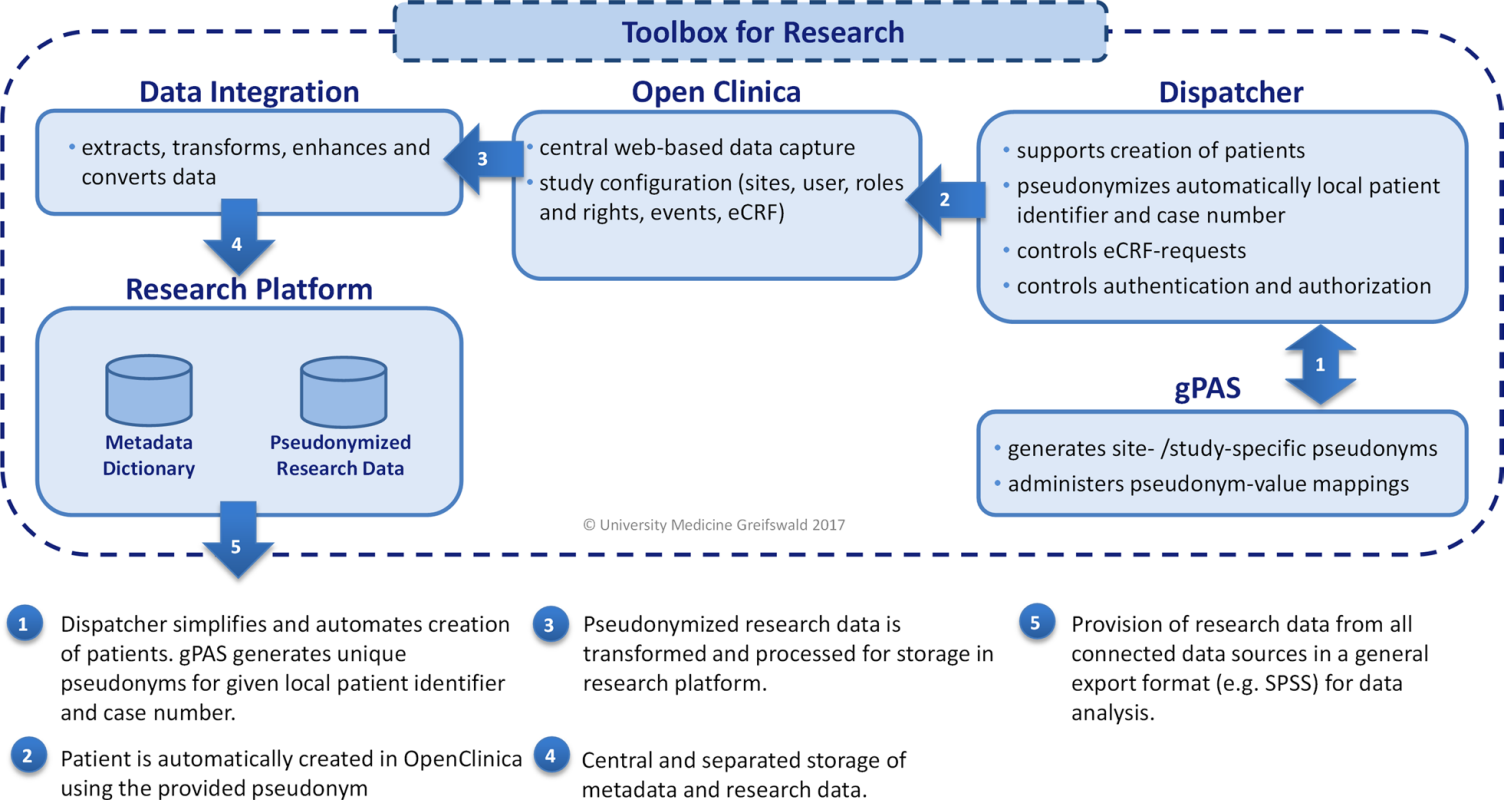
Process chain of the Toolbox for Research

Within the Toolbox a basic dispatcher module (following the concept of the Trusted Third Party Dispatcher [14] ) controls user authentication, administrates pseudonyms and simplifies the registration of new participants for a pre-configured study or event. In particular, after pseudonym generation the dispatcher redirects the data entry personnel directly to the data entry forms. The dispatcher also supports authorized study staff members to resolve patient ID (i.e. determine the used pseudonym or the combination of local patient identifier and case number), and, provides the necessary web forms (see Fig. 3).

### Container-based architecture

**Fig. 4:**
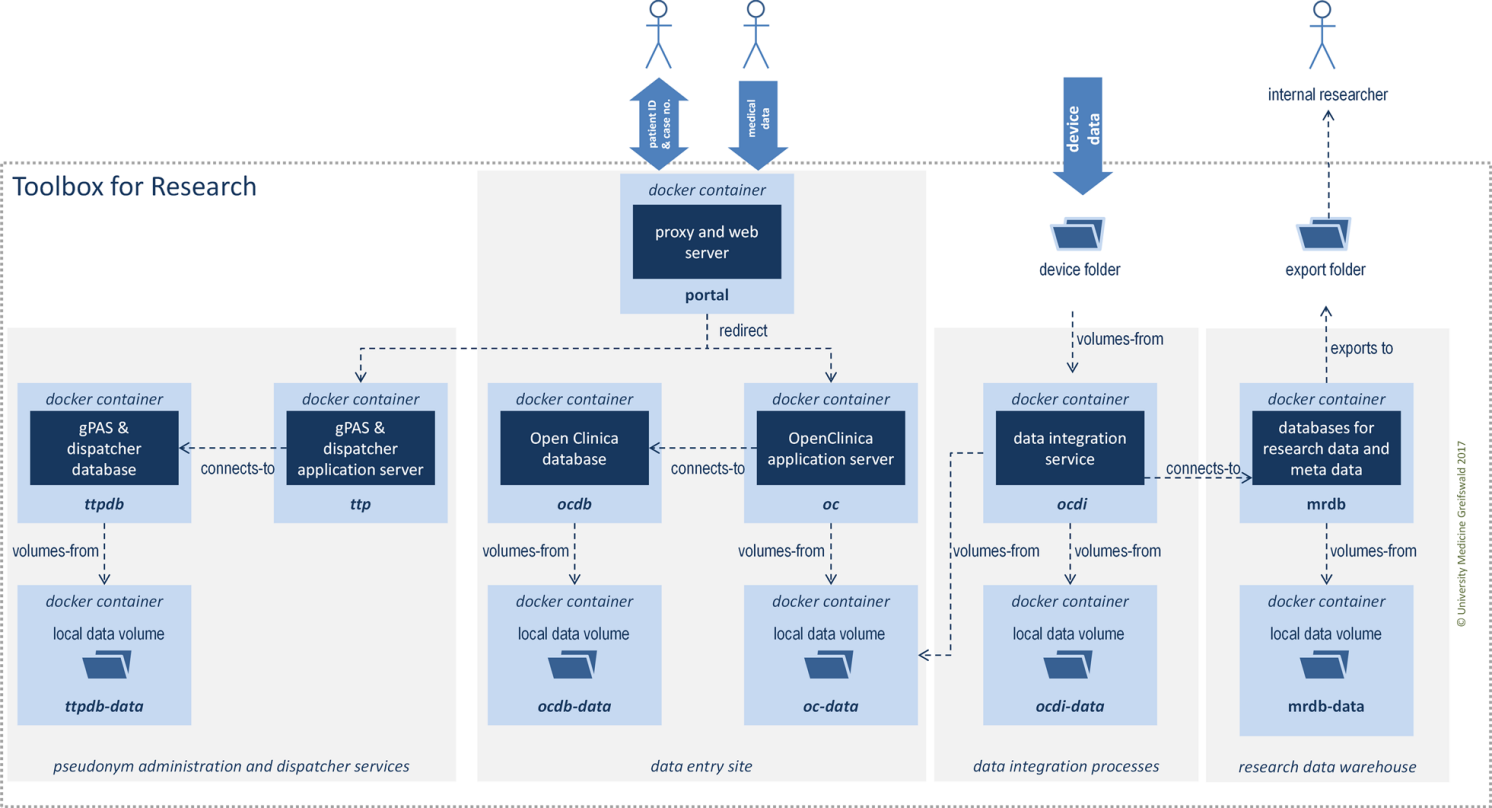
Architecture of the Toolbox for Research from a docker-container perspective

The Toolbox for Research uses Docker [16] containers to deploy the complex architecture as well as to separate systems and data (see Fig. 4) from each other. Additionally, essential configuration and data points are mounted in the */opt* directory of the host system. The containers communicate with each other via especially configured ports. Figure 4 provides an overview of the Docker architecture.

The separation of system and data containers simplifies system updates, maintainance and restoring processes. Additionally, it simplifies data protection processes. For example, the user can only enter data into the Toolbox for Research using an exclusive web front-end. Moreover, the access to selected databases is only possible via special ports with knowledge of the access credentials or only to explicitly permitted system areas such as data exports.

The installation process of the Toolbox for Research was fully automatized with installation scripts using Docker as the technical basis. The installation files as well as further documentation are available in the download area of the MOSAIC project [1] (https://mosaic-greifswald.de/werkzeuge-und-vorlagen/toolbox-for-research.html).

The Docker-based installation process of the Toolbox for Research is extensively documented (including installation, setup, operation, a checklist for administrators and a user manual) and simplified for non-IT experts [16]. This was considered necessary since Franklin et al. had identified documentation written in technical language as a barrier to using an EDC system. Moreover, the utilized central data management approach reduces maintenance efforts, e.g. software updates are applied to the centrally provided Toolbox server. Thus, participating research sites do not need to maintain local installations and always use the latest software version by accessing the centrally provided, web-based Toolbox.

## Results

The Toolbox for Research was developed by the Institute for Community Medicine of the University Medicine Greifswald as one part of the DFG-funded project MOSAIC. It provides a modular and re-usable open-source solution for a comprehensive central data management in epidemiological research projects of various sizes and application scenarios. Besides separated databases for metadata and research data, the Toolbox automates many essential technical processes (e.g. extraction, transformation and processing of research data as well as pseudonymization and data export). It enables the integration of individual modules such as quality assurance modules as well as several additional data sources (e.g. forms or devices). The Toolbox for Research also provides an OpenClinica system in order to be able to generate web-based eCRFs. Additionally, study sites, projects and users as well as their respective rights and roles are managed through OpenClinica. Furthermore, guidelines for developing a data dictionary as well as eCRFs are provided by the MOSAIC project in German and English language.

A data integration module and a dispatcher module were integrated into the Toolbox for Research. Both modules simplify Toolbox-internal processes, while an NGINX web server provides required web interfaces and file downloads for scientists. As an example, an export module was implemented, which aggregates metadata and research data at predefined intervals, and provides this aggregated data automatically in the SPSS format for analysis.

The Toolbox for Research addresses most of the mentioned requirements (see Table 1 and 2: requirement a1–a7) and comprises the following features:

- A generic data dictionary to support various application scenarios (according to Table 1, No. 1).
- An easy-to-use data protection compliant software solution facilitating a web-based pseudonymized data capture and, consequently, support of web-based eCRFs (according to Table 1, No. 1 and 2).
- Site-specific automatic generation of pseudonyms—therefore, no personally identifiable information are included in the Toolbox for Research-while at the same time ensuring traceability, if needed (according to Table 1, No. 3).
- Support of ETL processes by using automated processes (according to Table 1, No. 5).
- Separate storage of research and metadata (according to Table 1, No. 4).
- Standardized export of pseudonymized research data in SPSS format (according to Table 1, No. 6).
- User management and site-sensitive data management with the help of OpenClinica (according to Table 1, No. 7).
- An open-source EDC solution (according to Table 2, a1).
- Easy download from the MOSAIC project website and automatic installation via Docker (according to Table 2, a2).
- Optional integration of (laboratory or medical) device data (according to Table 2, a3).
- Adequate community support for OpenClinica and constant further development of the Toolbox based on user feedback (according to Table 2, a4).
- Detailed documentation for the Toolbox (including installation, setup, operation, a checklist for administrators and a user manual), which is simplified for non-IT experts to operate (according to Table 2, a7).

However, due to most complex technical and organizational frameworks, the Toolbox does not facilitate use and access procedures (according to Table 2, requirement a8) to share research data beyond the study context.

Since 1991 the German Society for Burn Treatment (Deutsche Gesellschaft für Verbrennungsmedizin e.V., DGV) and the working group “The severely burned child” (“Das schwerbrandverletzte Kind”) compile annual statistics based on data originating from burn centres as well as the inpatient treatment of children [17]. These annual statistics are centrally provided via the homepage of the DGV.

The German Burn Registry started in 2014 [7], and the total number of participating study sites as well as the set of variables increased continuously. Within the registry the burn treatment of patients was documented using MS Excel spreadsheets, which were manually merged for data analysis once a year. Thus, no central data repository existed and an automated integration of pseudonyms as well as additional information, e.g. from medical or laboratory devices, was not possible.

**Fig. 5:**
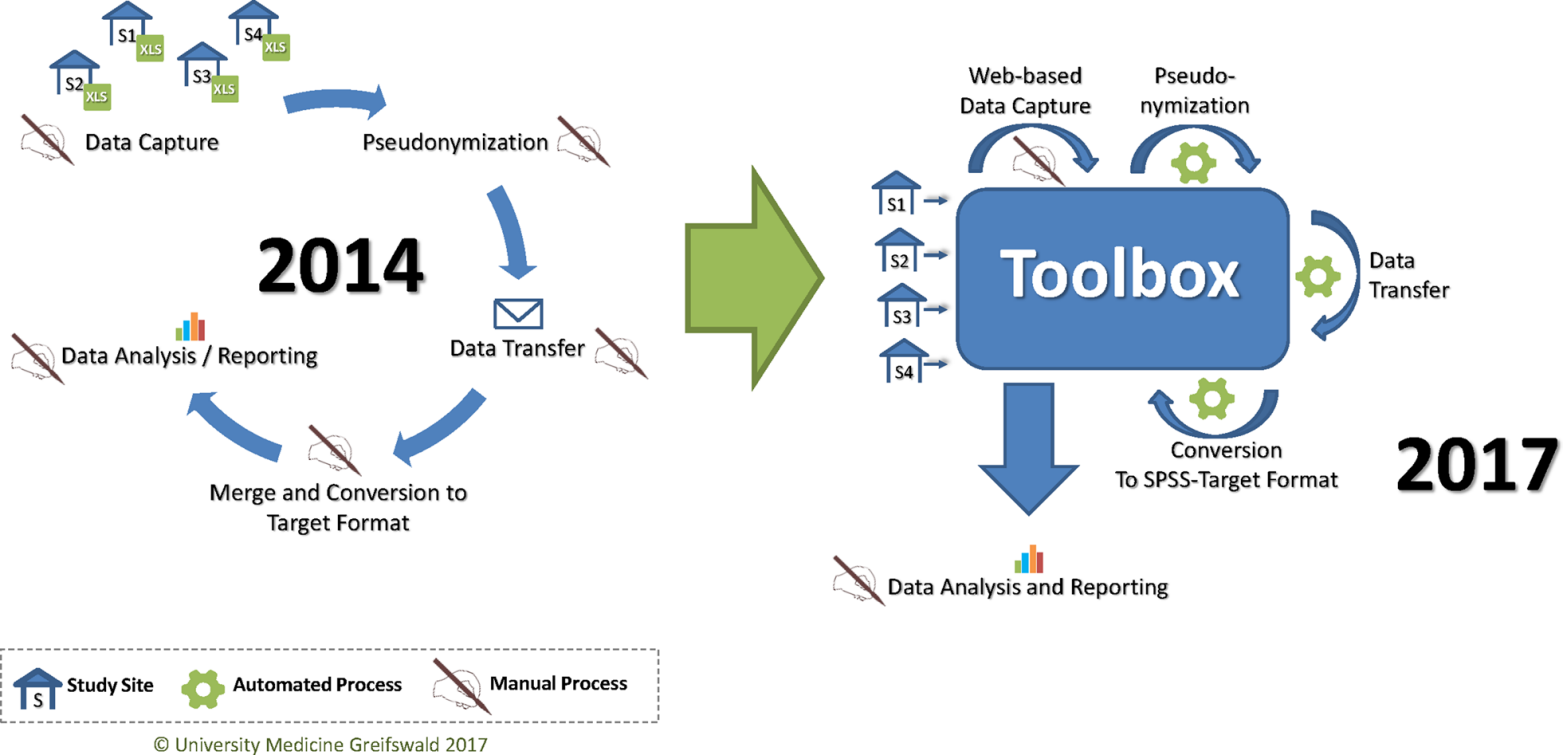
Comparison of manual and automated data management processes within the German Burn Registry in the years 2014 and 2017

In January 2016 the German Burn Registry officially replaced the annual statistics of the DGV [17] and as a proof of concept, the Toolbox for Research was used for a technical upgrade of the internal data management processes (cf. Fig. 5).

**Fig. 6:**
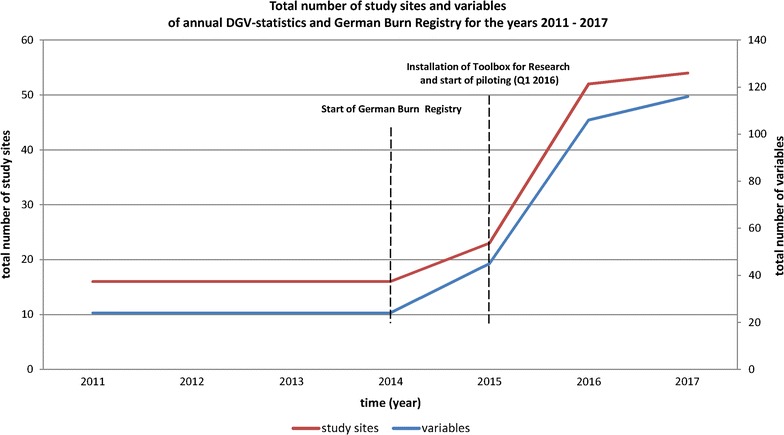
Total number of study sites and variables of annual DGV-statistics and the German Burn Registry for the years 2011–2017

Data capture and, thus, piloting the Toolbox for Research officially started in April 2016. As of April 11th, 2017, approximately 1 year after the start of this pilot phase, the German Burn Registry had gathered data of 4975 patients at 54 sites. Within the German Burn Registry essential data management processes, formerly handled manually, could be successfully automated, as depicted in Fig. 5. At the same time, the total number of variables and registered study sites could be increased significantly (cf. Fig. 6). As a result the total number of documented burn cases per year could be extended considerably (2014: 1.408 cases; 2016: 4.350 cases). Therefore, the pilot phase could be successfully concluded and the implemented Toolbox solution will be continuously used within the German Burn Registry.

The Toolbox for Research is provided as free of charge, open-source EDC solution and can be downloaded from the website of the MOSAIC project [18] . The Toolbox is part of the ToolPool for Medical Research (powered by the TMF e.V.) [19] .

## Discussion

Our Toolbox for Research ensures compliance with data protection requirements with creating site- and study-specific pseudonyms centrally by the pseudonym administration service gPAS. It also enables authorized personnel to search for pseudonyms and de-pseudonymize study participants if necessary. For example, the follow-up of study participants (e.g. when changing locations) can use a “previous registry pseudonym”. The integrated dispatcher module supports an easy creation and registration of study participants. Additionally, the utilization of OpenClinica enables the user to generate eCRFs as well as web-based and multi-site data collection using the Toolbox for Research guidelines and OpenClinica mechanisms. Furthermore, the Toolbox for Research facilitates the separate storage of meta- and research data as well as the integration of device data (for example in CSV format). Data exports are currently provided in the SPSS format.

However, the Toolbox for Research does not automatically define a data dictionary or eCRFs. Throughout the interactive steps the user can refer to guidelines provided with the system. Additionally, the Toolbox ensures basic data protection by pseudonymizing research data. Personally identifiable information are not stored within the Toolbox for Research.

However, the Toolbox cannot provide nor substitute functionalities of a Trusted Third Party [14] . Since only case numbers or patient IDs (without further personally identifiable information) are stored within the Toolbox, checks for duplicates cannot be conducted. Only birthdate and sex are known and stored as research data because both parameters have to be entered to register a participant within OpenClinica. At present, automatic data analyses, quality monitoring processes or evaluation of the data dictionary’s scientific quality are not supported within the Toolbox for Research.

The Toolbox for Research provides an integrated OpenClinica (v.3.4) system. Consequently, known OpenClinica limitations regarding the development of eCRFs arise. For example, conditional jumps, plausibility checks across more than one variable as well as validation of entered data are not possible without additional JavaScript competence.

Even though the use and installation of the Toolbox for Research does not require specific IT knowledge and its aim is to support researchers and non-IT experts, the Toolbox requires the user to provide and secure the necessary IT infrastructures. For example, to be able to run the Toolbox for Research a mixed model is necessary to have the necessary root rights while still getting support in securing and operating the system (as well as e.g. user management and creation of eCRFs). The relevant issues were collected and are addressed in a checklist for administrators (e.g. data backup, ensuring secure web access (DNS and certificates), user authentication as well as maintenance and monitoring of the system). Consequently, a certain level of IT support is still required to use the Toolbox for Research.

## Conclusions

The objectives of the Toolbox for Research developed by the Institute for Community Medicine of the University Medicine Greifswald within the MOSAIC project [1] were to support researchers with their research projects in different scenarios by providing a flexible and easy to use software solution. Consequently, all identified requirements (see Tables 1, 2) for a comprehensive data management in research should be considered. Thus, the Toolbox for Research is easily accessible via the MOSAIC project website and is free of charge.

Since the Toolbox for Research focusses on researchers with limited IT resources and competences, the installation processes were automated and extensive user manuals are provided, e.g. for configuring and operating the Toolbox. Additionally, guidelines and templates for developing the necessary DD and eCRFs are provided in German and English.

As a proof of concept, the Toolbox for Research was successfully established in the German Burn Registry [7], which piloted the Toolbox over a 1-year period with 173 active users (2017). Today, the Toolbox for Research is an extensive web solution for data capture that can help to replace general-purpose application software like spreadsheets in small-scale research studies and registries.

Most problems during the pilot phase resulted from limitations of OpenClinica, which had to be solved by additional implementations using JavaScript. For example, calculating time periods between two dates or timestamps as well as calculating the body mass index had to be implemented using JavaScript. Performing plausibility checks to ensure that all entered values were in the correct format before entering the calculations or a check, that at least one answer option of an eCRF item was selected, also had to be implemented manually. OpenClinica does allow conditional displays for checkboxes or radiobuttons only, but those are not applicable for value-based checks. Furthermore, missing values can only be included with non-metric variables, if a validation is to be performed without regular expressions. However, OpenClinica is only integrated into the Toolbox for Research as an examplary implementation of an EDC system. With additional effort any other open-source EDC system can also be used.

Some modules of the Toolbox for Research are also separately available. The gPAS is continuously under further development. Potential next steps are the integration of the R package MOQA [20] [ into the Toolbox, and runtime optimizations. Additionally, authentication will be enhanced, since only basic authentication is available at the moment.

## Abbreviations

D2: data dictionary
DD: data dictionary
DNS: Domain Name Service
eCRF: Electronic Case Report Form
EDC: electronic data capture
ETL: extract transform load
gPAS: generic pseudonym administration service
IT: information technology
MDAT: medical research data
OCDI: OpenClinica Data Integration
PII: personally identifiable information
R2: research repository
